# Facile Enhancement of Electrochemical Performance of Solid-State Supercapacitor via Atmospheric Plasma Treatment on PVA-Based Gel-Polymer Electrolyte

**DOI:** 10.3390/gels9040351

**Published:** 2023-04-21

**Authors:** Dong-Hyun Kim, Suk Jekal, Chan-Gyo Kim, Yeon-Ryong Chu, Jungchul Noh, Min Sang Kim, Neunghi Lee, Woo-Jin Song, Chang-Min Yoon

**Affiliations:** 1 Department of Chemical and Biological Engineering, Hanbat National University, Daejeon 34158, Republic of Korea; 2 McKetta Department of Chemical Engineering and Texas Material Institute, The University of Texas at Austin, Austin, TX 78712, USA; 3 Department of Polymer Science and Engineering, Chungnam National University, Daejeon 34134, Republic of Korea; 4 Department of Chemical Engineering and Applied Chemistry, Chungnam National University, Daejeon 34134, Republic of Korea; 5 Department of Organic Materials Engineering, Chungnam National University, Daejeon 34134, Republic of Korea

**Keywords:** atmospheric plasma treatment, PVA, hydrophilicity, solid-state supercapacitor, gel-polymer electrolyte

## Abstract

A facile oxygen (O_2_) atmospheric plasma treatment is applied to a polyvinyl alcohol (PVA) matrix to enhance its wettability and hydrophilicity. The optimal plasma treatment conditions are determined by varying the applied plasma power and plasma treatment time. A PVA matrix treated with a plasma power of 120 W for 5 s shows the most hydrophilicity owing to successful formation of carbonyl (–CO, >C=O) functional groups without any structural degradation. The plasma-treated PVA matrix is used as the gel-polymer electrolyte of a solid-state supercapacitor (SSC) by immersing solid matrix into various liquid electrolytes, such as sodium sulfate (Na_2_SO_4_), sulfuric acid (H_2_SO_4_), and potassium hydroxide (KOH). Compared with the pristine PVA-based device, PVA-120W5/Na_2_SO_4_-, PVA-120W5/H_2_SO_4_-, and PVA-120W5/KOH-based SSCs show 2.03, 2.05, and 2.14 times higher specific capacitances, respectively. The plasma-treated PVA matrix shows increased specific capacitance owing to the increased wettability, which in turn increases the ion transportation and reduces the electrical resistance. This study successfully demonstrates that the electrochemical performance of a SSC can be readily enhanced through plasma treatment for a short time (≤5 s).

## 1. Introduction

Energy storage devices, such as supercapacitors (SCs) and batteries, are widely used in hybrid and electric vehicles, renewable energy systems, and various electronic devices [1,2]. SCs provide several benefits over batteries, such as rapid charge/discharge rates, long-term cyclability, and high power density [3]. In particular, solid-state supercapacitors (SSCs) are expected to be used in future energy storage devices that are increasingly demanded for flexible, wearable, and portable devices [4]. An SC typically consists of two electrodes (one positive and one negative), a separator, and an electrolyte [5]. Various materials including carbon, transition metal oxides, and conductive polymers can be used as active materials for electrodes [6]. The electrolyte determines the interfacial resistance at the electrode interface, as well as the ionic conductivity, and thus, it is a key factor affecting the SC performance [7,8]. SSCs employ gel or solid electrolytes instead of liquid ones because they offer superior mechanical durability, thermal stability, outstanding electrochemical performance, and a flexible form factor [9,10].

In SSCs, various materials such as polymers, ceramics, and composite materials have been used as gel electrolytes [11,12]. Among them, gel-polymer electrolytes, with their advantages over liquid electrolytes, such as mechanical stability, high ion conductivity, and safety, have received widespread attention as a key component in electrochemical energy storage devices, including SCs and batteries [13,14,15]. Polymers, such as polyvinyl alcohol (PVA), polyvinylidene fluoride (PVDF), polyolefin, and polymethyl methacrylate (PMMA), are used most commonly, because of their chemical stability, ease of production, and low cost [16,17,18,19]. When dissolved in a water-soluble solvent, polymers can be gelated to form a gel-polymer electrolyte [20,21]. In particular, PVA is widely used for synthesizing gel-polymer electrolytes owing to its high ionic conductivity, flexibility, mechanical/thermal stability, and nontoxicity [22,23,24]. Moreover, PVA can be simply modified by introducing other functional groups, making it a versatile material for gel-polymer electrolyte applications [25]. The functional groups of PVA-based electrolytes can be conveniently adjusted using various physical and chemical techniques, including plasma treatment [26,27].

Plasma treatment is a simple process in which the surface properties of a material are modified using ionized gas without impacting the bulk properties [28,29]. By selecting appropriate gases, desired characteristics including wettability, adhesion, and biocompatibility can be conferred on the material surface [30,31]. Plasma treatment can be performed in a vacuum or using atmospheric plasma [32]. In a vacuum, the low pressure allows the plasma to be modified and functionalized more precisely by using various gases, such as oxygen (O), nitrogen (N), and fluorine (F) [33,34,35]. Compared with vacuum plasma, atmospheric plasma affords advantages, such as ease of operation, quickness, safety, and mass production [36,37]. Furthermore, atmospheric plasma activates near atmospheric pressure, making the process more convenient and less expensive to operate [38].

In particular, O_2_ atmospheric plasma treatment has attracted much attention because it can effectively enhance the wettability and adhesion of a target material [39,40]. Inherently hydrophobic materials, such as polymers, can be made hydrophilic by introducing hydroxyl (–OH) and carbonyl (–CO and >C=O) functional groups generated by O_2_ atmospheric plasma treatment [41,42]. In electrochemistry, surface modification is considered highly important because low wettability or adhesion of the active material, electrode, electrolyte, and separator can cause a high electrical resistance that degrades the electrochemical performance [43]. Some studies have investigated the enhancement of separator adhesion to electrodes in secondary batteries via O_2_ plasma treatment. For instance, Li et al. successfully generated various electronegative oxygenic functional groups such as carbonyl and hydroxyl on the separator surface via O_2_ plasma treatment and thereby enhanced the electrochemical performance of a Li-S battery [44]. Jin et al. improved the adhesion between a polyethylene separator and an electrode via the surface modification of the separator by O_2_ plasma treatment and thereby enhanced the electrochemical performance of a Li battery [45]. Such treatment promotes the movement of ions and ensures stable adhesion with the electrode to lower the electrical resistance, thereby improving the overall battery performance [46,47]. However, most of plasma treatment employed electrochemical studies were focusing on the fields of secondary batteries. There were some studies regarding the enhancement of electrochemical performance of supercapacitors, but previous studies were concentrated in the treatment on active material and electrode [48,49,50]. In this regard, there is necessity for a better understanding of plasma effects and related electrochemical performance on gel or solid electrolytes.

In this study, a PVA-based gel-polymer electrolyte (pristine PVA) was synthesized by the simple gelation of a PVA solution. To improve the electrochemical performance, the surface of the gel-polymer electrolyte was then functionalized through fast and effective O_2_ atmospheric plasma treatment (plasma-treated PVA). By controlling the intensity of the applied plasma power from 80 to 160 W (referred to as PVA-80W5, PVA-100W5, PVA-120W5, PVA-140W5, and PVA-160W5) and the treatment time from 5 to 25 s (referred to as PVA-120W5, PVA-120W10, PVA-120W15, PVA-120W20, and PVA-120W25), an applied power of 120 W and treatment time of 5 s (PVA-120W5) were identified to be the most effective conditions for introducing oxygen functional groups without causing polymer decomposition. The SSCs were then assembled using C/MnO_2_ and C/Fe_2_O_3_ as the positive and negative electrodes, respectively. Next, 1.0 M Na_2_SO_4_ was applied to the plasma-treated PVA (PVA-120W5/Na_2_SO_4_). The PVA-120W5/Na_2_SO_4_-based SSC showed a specific capacitance of ca. 7.89 mF cm^−2^ at 100 mV s^−1^; this was 2.03 times higher than that of a pristine PVA/Na_2_SO_4_-based device (i.e., 3.89 mF cm^−2^). The plasma-treated PVA matrix was further immersed into liquid electrolytes of 1.0 M H_2_SO_4_ and 1.0 M KOH. The PVA-120W5/H_2_SO_4_- and PVA-120W5/KOH-based SSCs exhibited 2.05 and 2.14 times higher capacitances, respectively, than that of their corresponding pristine PVA-based devices. In this manner, hydrophilic functional groups were successfully introduced to the PVA-based gel-polymer electrolyte using facile O_2_ atmospheric plasma treatment within a short time (≤5 s), and consequently, the electrochemical performance for SC applications was enhanced.

## 2. Results and Discussion

### 2.1. Fabrication of Plasma-Treated PVA-Based Gel-Polymer Electrolyte (Plasma-Treated PVA)

**Figure 1:**
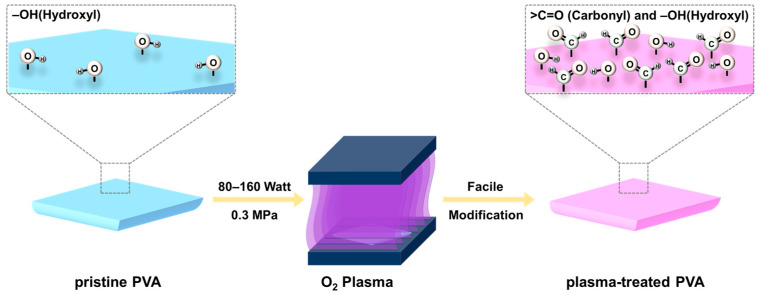
Schematic illustration of the synthesis of plasma-treated PVA-based gel-polymer electrolyte (plasma-treated PVA) through O_2_ atmospheric plasma treatment.

Figure 1 illustrates the process of O_2_ atmospheric plasma treatment of pristine PVA. The pristine PVA was fabricated via a simple gelation method. PVA powder was dissolved in deionized (DI) water with vigorous stirring to form a gel-like solution. PVA can be dissolved in a water-soluble solvent owing to the presence of hydrophilic groups, such as –OH (hydroxyl). Subsequently, pristine PVA was successfully synthesized by drop-casting the PVA solution followed by a drying process. O_2_ plasma treatment was then performed to enhance the wettability of pristine PVA by introducing hydrophilic groups, such as –CO and >C=O (carbonyl groups). The applied plasma power was varied in the range of 80–160 W for 5 s to determine the appropriate condition for introducing the hydrophilic groups. After confirming the applied plasma power, the treatment time was varied in the range of 5–25 s to determine the optimal condition for enhancing the wettability.

**Figure 2:**
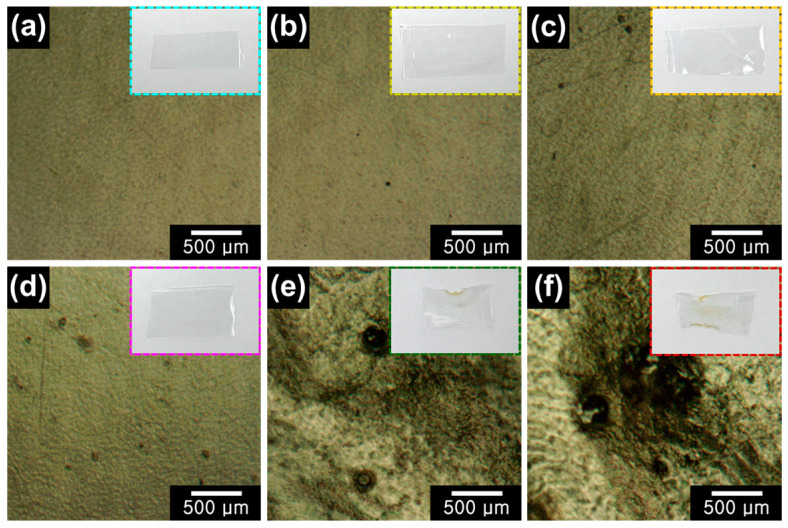
Optical microscope (OM) images of (**a**) pristine PVA, (**b**) PVA-80W5, (**c**) PVA-100W5, (**d**) PVA-120W5, (**e**) PVA-140W5, and (**f**) PVA-160W5 (inset: corresponding digital photograph).

Optical microscopy (OM) analysis was conducted to investigate the surface conditions of pristine and various plasma-treated PVA matrices (Figure 2). In case of pristine PVA, flat surface was observed without any damages [51]. On the other hand, damaged surfaces by thermal deformation were clearly discovered for all plasma-treated PVA matrices. In specific, thermal energies of plasma treatment have left the brownish scorched damages on the surface and damages have increased with the applied power of plasma [52]. Additionally, scanning electron microscopy (SEM) was conducted to verify the surface conditions of each plasma-treated PVA (Figure S1). Similar to the results of OM images, surface damages were observed for the various plasma-treated PVA matrices.

**Figure 3:**
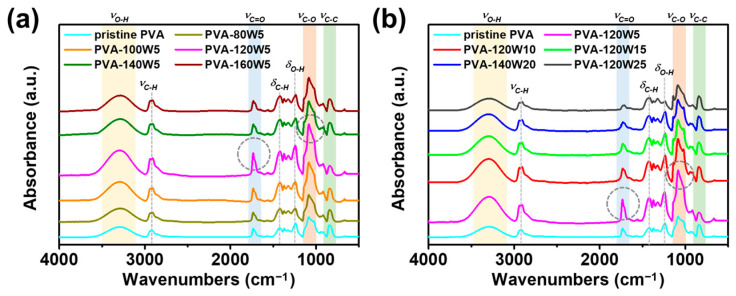
FT-IR spectra of pristine and plasma-treated PVA matrices with varying (**a**) plasma power in the range of 80–160 W (plasma treatment time: 5 s), and (**b**) plasma treatment time in the range of 5–25 s (applied plasma power: 120 W).

Fourier-transform infrared (FT-IR) analysis was conducted for pristine and plasma-treated PVA matrices to further investigate the changes in their molecular composition after plasma treatment (Figure 3). First, pristine and plasma-treated PVA with applied plasma powers of 80–160 W for 5 s were examined, as shown in Figure 3a. In all cases, characteristic peaks corresponding to the O–H, C=O, and C–O stretching vibrations and C–C stretching vibration were observed at 3290, 1730, 1086, and 842 cm^−1^, respectively [53,54,55,56]. Additionally, minor peaks with low intensities were detected for C–H stretching, C–H bending, and O–H bending at 2932, 1424, and 1325 cm^−1^, respectively [56,57,58]. As the applied plasma power was increased up to 120 W, the absorbance of hydrophilic groups such as O–H, C=O, and C–O increased whereas that of the C–C group decreased [59,60]. As the applied plasma power was increased beyond 120 W, the absorbance of hydrophilic groups decreased, suggesting the structural deformation of the PVA matrix. Based on the FT-IR analysis, the optimal power for the surface modification of PVA through plasma treatment were determined to be 120 W.

To further optimize the condition of most hydrophilic functional groups, the plasma treatment time was varied as 5, 10, 15, 20, and 25 s (listed as PVA-120W#seconds), while maintaining the applied plasma power of 120 W (Figure 3b). The absorbance of O–H, C=O, and C–O groups was the highest when the plasma treatment time was 5 s. As the plasma treatment time was increased beyond 5 s, the absorbance of hydrophilic groups decreased, causing the structural deformation of PVA. Therefore, the optimal conditions to prepare the most hydrophilic plasma-treated PVA matrix were determined to be an applied plasma power of 120 W and treatment time of 5 s.

**Figure 4:**
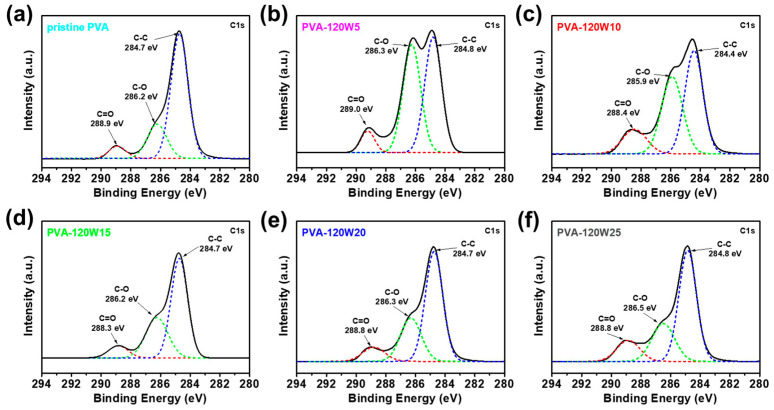
XPS C 1s spectra of (**a**) pristine PVA, (**b**) PVA-120W5, (**c**) PVA-120W10, (**d**) PVA-120W15, (**e**) PVA-120W20, and (**f**) PVA-120W25.

X-ray photoelectron spectroscopy (XPS) analysis was performed to investigate the molecular characteristics of pristine and plasma-treated PVA matrices with plasma treatment times of 5–25 s, as shown in Figure 4. As discussed previously, the optimized plasma treatment power of 120 W resulted in the most hydrophilic functional groups without structural deformation. In the C 1s spectra, C–C, C–O, and C=O bonds were detected at approximately 284.7, 286.9, and 289.0 eV for all samples, respectively [61]. In pristine PVA, the intensity of the C–C peak dominated those of carbonyl groups such as C–O and C=O. In plasma-treated PVA matrices, the peak intensities of carbonyl groups increased whereas that of C–C decreased. Notably, hydrophilic groups with the highest intensities were observed in case of PVA-120W5. As the plasma treatment time was increased beyond 5 s, the intensities of hydrophilic groups started to decrease because the initiation of the PVA etching process caused the deformation of the material [62]. Through the plasma treatment of pristine PVA, the optimal conditions for introducing the most hydrophilic functional groups without destroying the molecular structures were determined to be an applied plasma power of 120 W and treatment time of 5 s.

**Figure 5:**
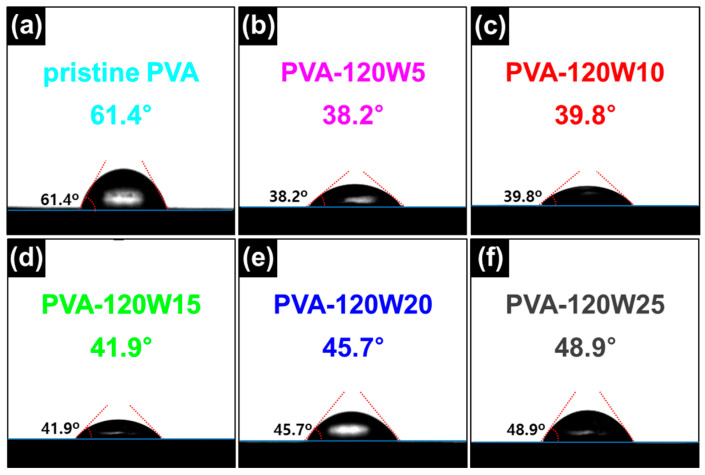
CA images of water droplet on surface of (**a**) pristine PVA, (**b**) PVA-120W5, (**c**) PVA-120W10, (**d**) PVA-120W15, (**e**) PVA-120W20, and (**f**) PVA-120W25.

Contact angle (CA) measurements were performed to evaluate the wettability change according to the plasma treatment time with an applied plasma power of 120 W (Figure 5). The measured CA for pristine PVA, PVA-120W5, PVA-120W10, PVA-120W15, PVA-120W20, and PVA-120W25 was approximately 61.4, 38.2, 39.8, 41.9, 45.7, and 48.9°, respectively. Relative to the pristine PVA, all plasma-treated PVAs showed a lower CA owing to the formation of hydrophilic functional groups. However, the CA values increased with an increase in the plasma treatment time. This increase in CA is caused by the thermal degradation of functional groups by plasma treatment [63]. Specifically, the plasma treatment functionalized the PVA surface; however, continuous plasma treatment can cause the degradation and dissociation of the carbonyl functional groups. Therefore, an applied plasma power of 120 W and treatment time of 5 s (PVA-120W5) were determined as the optimal conditions to prepare the most hydrophilic PVA matrix.

In addition, swelling ratios of various plasma-treated PVAs were determined to further investigate the plasma effect on the wettability. Swelling ratios of pristine and plasma-treated PVAs were determined by immersing the sample into the solution for 10 s and determining the mass ratio between initial dry state and wetted state. Determined swelling ratio of Pristine PVA, PVA-120W5, PVA-120W10, PVA-120W15, PVA-120W20, and PVA-120W25 in aqueous solution were 2.3, 3.6, 3.3, 3.3, 3.2, and 2.7, respectively. It was confirmed that the swelling ratio of various plasma-treated PVAs were in accordance with the contact angle measurements. Additionally, the swelling ratios were measured for two organic solvents EtOH and commercial EC/EMC 3:7 (1.0M LiPF_6_) electrolyte. In case of EtOH, the evaluated swelling ratios were determined as 1.7, 2.6, 2.4, 2.3, 2.3, and 1.9, showing similar trends to aqueous solution. For the commercial EC/EMC 3:7 electrolyte (1.0M LiPF_6_), determined swelling ratios were 1.6, 1.8, 1.6, 1.3, 1.4, and 1.1. Notably, swelling trends of various plasma-treated PVAs were similar in two organic solvents; this led to a maximized swelling ratio in PVA-120W5 condition, decreasing after the plasma treatment times of 5 s. Polarities of organic solvents were lower compared to water or aqueous solution, but the presence of functional groups of EtOH and EC/EMC provided a binding effect with the functional groups of pristine and plasma-treated PVA matrices [64,65]. In this regard, swelling ratios were different for each solvent, but overall trends were similar due to the functional groups. After the 5 s of plasma treatment decrement in swelling ratio was observed due to the degradation of carbonyl functional groups. Accordingly, organic solvent with polar atom or functional groups manifested a similar uptake trend to aqueous solution. Detailed swelling ratios of pristine and plasma-treated PVAs after immersing in aqueous solution, EtOH, and organic solution are listed in Table S1.

### 2.2. Electrochemical Properties of Plasma-Treated PVA-Based SSC

Cyclic voltammetry (CV) measurements and electrochemical impedance spectroscopy (EIS) were used to investigate the improvement in the electrochemical performance of plasma-treated PVA. The SSC was assembled using C/MnO_2_ as the positive electrode, C/Fe_2_O_3_ as the negative electrode, and plasma-treated PVA matrix with added Na_2_SO_4_ as the gel-polymer electrolyte. MnO_2_ and Fe_2_O_3_ were mixed with the activated carbon material, owing to their large work function difference, high electrochemical performance, and hydrophilicity [66,67]. Furthermore, the pristine and plasma-treated PVA were immersed in 1.0 M Na_2_SO_4_ and employed as gel-polymer electrolytes to verify the difference in their electrochemical performance without destroying the electrode during the charging and discharging process [68].

**Figure 6:**
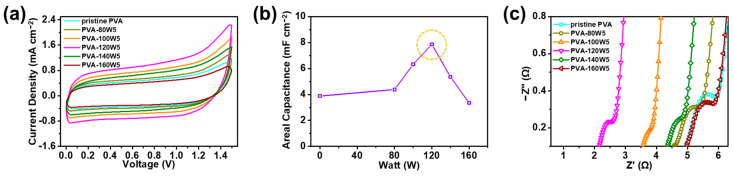
(**a**) CV curves of SSCs employing plasma-treated PVA matrices with different plasma powers at a scan rate of 100 mV s^−1^. (**b**) Areal capacitances evaluated from CV curves for SSCs employing plasma-treated PVA matrices with different plasma powers. (**c**) EIS results of SSCs employing plasma-treated PVA matrices with different plasma powers.

First, pristine and plasma-treated PVA with different plasma treatment powers were analyzed according to the CV curves at a scan rate of 100 mV s^−1^ in the potential window of 0–1.5 V, as shown in Figure 6a. The areal specific capacitance evaluated from the CV curves was 3.89, 4.36, 6.37, 7.89, 5.33, and 3.31 mF cm^−2^ for pristine PVA, PVA-80W5, PVA-100W5, PVA-120W5, PVA-140W5, and PVA-160W5, respectively (Figure 6b). Notably, PVA-120W5 exhibited the highest areal specific capacitance, in keeping with the hydrophilicity results obtained through FT-IR analysis. When the applied power was increased beyond 140 W, the areal capacitance decreased, suggesting the structural decomposition of PVA. The introduction of hydrophilic groups using facile plasma treatment increased the wettability of the gel-polymer electrolyte, which was capable of retaining its wettability and a large number of ions, resulting in the enhancement in electrochemical performance [69,70].

EIS analysis of the pristine and plasma-treated PVA matrices was used to compare the ion-transport capabilities of the as-assembled SSCs, as shown in Figure 6c. The corresponding equivalent circuit of the SSC is shown in Figure S2; the circuit consists of an equivalent circuit resistance (*R*_ESR_), charge transfer resistance (*R*_CT_), Warburg impedance (*Z*_W_), and double-layer capacitance (*C*_DL_) [71]. Each EIS data was investigated by fitting the data into an equivalent circuit model and adding the fitted curve as an inset, as shown in Figure S5. *R*_ESR_ is the ionic resistance of the electrolyte and the interface resistance of the active material [72,73]. *R*_CT_ is the resistance of the faradaic reaction at the electrolyte–electrode interface [74]. For pristine PVA, PVA-80W5, PVA-100W5, PVA-120W5, PVA-140W5, and PVA-160W5, *R*_ESR_ values were measured to be 4.91, 4.59, 3.45, 2.12, 4.30, and 4.90 Ω, respectively, and the corresponding *R*_CT_ values were measured to be 1.46, 1.20, 0.84, 0.80, 1.06, and 1.32 Ω, respectively. PVA-120W5 showed the lowest *R*_CT_ and *R*_ESR_ values of 0.80 and 2.12 Ω, respectively. MnO_2_ and Fe_2_O_3_ from each electrode are hydrophilic and therefore effectively activate ion transportation at the electrode–electrolyte interface more dynamically to reduce *R*_CT_ [75,76]. Therefore, SSCs employing PVA-120W5 exhibited the highest capacitive behavior.

**Figure 7:**
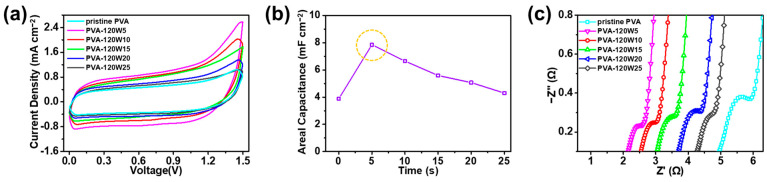
(**a**) CV curves of SSC devices using plasma-treated PVA matrices with different treatment times at a scan rate of 100 mV s^−1^. (**b**) Areal capacitances calculated from CV curves for SSCs using plasma-treated PVA matrices with different treatment times. (**c**) EIS analysis of SSCs using plasma-treated PVA matrices with different treatment times.

Moreover, to investigate the effect of the plasma treatment time on the electrochemical performance, CV curves of SSCs using plasma-treated PVAs with various treatment times of 5–25 s were measured at a scan rate of 100 mV s^−1^, as shown in Figure 7a. The PVA-120W5-based SSC had the largest area, indicating the highest electrochemical performance. The areal specific capacitances of SSCs using PVA-120W5, PVA-120W10, PVA-120W15, PVA-120W20, and PVA-120W25 were calculated to be 7.89, 6.77, 5.91, 5.13, and 4.36 F cm^−2^, respectively (Figure 7b). As the plasma treatment time of the electrolyte increased, the ability to retain water decreased, owing to the decrease in the hydrophilic groups of PVA, in turn resulting in a decrease in the ionic conductivity.

EIS analysis was conducted to verify the difference in the electrochemical performance of plasma-treated PVA according to the plasma treatment time, as shown in Figure 7c. In addition, the detailed information of each EIS data was further stated in Figure S4. For PVA-120W5, PVA-120W10, PVA-120W15, PVA-120W20, and PVA-120W25-based SSCs, the *R*_ESR_ values were measured to be 2.12, 2.47, 2.99, 3.66, and 4.21 Ω, respectively, and the corresponding *R*_CT_ values were measured to be 0.80, 0.92, 1.00, 1.18, and 1.21 Ω, respectively. The *R*_ESR_ and *R*_CT_ values increased with the plasma treatment time, confirming the increase in interface resistance between the electrode and electrolyte and decrease in ionic conductivity. Such phenomena occurred because the carbonyl group formed in PVA disintegrated as the plasma treatment time was increased beyond 5 s [77]. Therefore, PVA-120W5 showed improved hydrophilicity that effectively controlled the interface resistance and ionic conductivity, thus confirming its suitability as a gel-polymer electrolyte for the SSC device.

**Figure 8:**
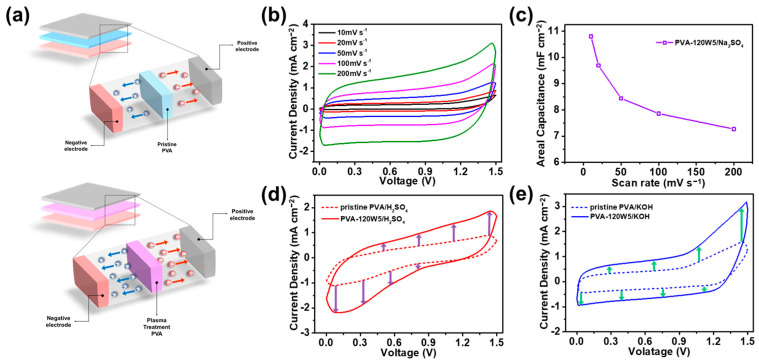
(**a**) Tentative mechanism of increased ionic conductivity of SSCs using plasma-treated PVA matrix. (**b**) CV curves of SSCs using PVA-120W5/Na_2_SO_4_ at different scan rates. (**c**) Areal capacitances of SSCs calculated from CV curves at different scan rates. (**d**) CV curves of SSCs using PVA-120W5/H_2_SO_4_ and (**e**) PVA-120W5/KOH before and after plasma treatment.

Figure 8a shows a plausible mechanism by which the ionic conductivity of SSCs using a plasma-treated PVA matrix increases, resulting in improved electrochemical performance. Compared with pristine PVA, plasma-treated PVA has more hydrophilic functional groups that can transport ions more conveniently. Figure 8b shows CV curves of the SSC using PVA-120W5/Na_2_SO_4_ at different scan rates of 10–200 mV s^−1^. The CV curves of the SSC maintained a rectangular shape even at high scan rates, suggesting the excellent ion transportation and capacitive properties [78,79]. In addition, the areal specific capacitances of the SSC measured from the CV curves were 10.77, 9.68, 8.41, 7.89, and 7.27 mF cm^−2^ at 10, 20, 50, 100, and 200 mV s^−1^, respectively (Figure 8c). Specifically, the capacitance retention was 67% as the scan rate was increased from 10 to 200 mV s^−1^, verifying the outstanding capacitive performance at even high scan rates [80,81]. Therefore, PVA-120W5 demonstrated improved performance as an SSC with ions and water being retained effectively by introducing hydrophilic groups using atmospheric plasma treatment. In addition, a cyclability test was conducted on the SSCs to compare the difference in retention rates between pristine PVA and PVA-120W5 at a current density of 5 mA cm^−2^ for 1000 cycles (Figure S7). The specific capacitances of the SSC devices employing pristine PVA and PVA-120W5 were evaluated as ca. 71.4 and 83.0% of their initial values, which verified the long-term stability of the SSC device. Furthermore, the CV curves of an SSC using PVA-120W5 with 1.0 M H_2_SO_4_ and 1.0 M KOH were examined at a scan rate of 100 mV s^−1^ to investigate the performance improvement with other electrolytes; CV curves obtained with the application of pristine PVA are shown for comparison (Figure 8d,e). The SSCs using PVA-120W5/H_2_SO_4_ and PVA-120W5/KOH exhibited 2.05 and 2.14 times higher electrochemical performance, respectively, compared with that of the SSC using pristine PVA. These results confirmed that introducing plasma-treated PVA increased the ionic conductivity and thereby enhanced the performance of SSCs. The newly prepared PVA-120W5/Na_2_SO_4_ was compared with other plasma-treated materials in SCs and summarized in Table 1 [48,82,83]. All other studies have reported on the enhancement of electrochemical performance by plasma treatment on active material and electrode. Accordingly, it was verified that the plasma treatment on various target or component including gel electrolyte can effectively enhance the electrochemical performance of supercapacitors.

## 3. Conclusions

Pristine PVA was subjected to O_2_ atmospheric plasma treatment. SSCs using the plasma-treated PVA exhibited improved electrochemical performance. PVA offers high ionic conductivity, and, therefore, it was used as a gel-polymer electrolyte via a simple gelation method. O_2_ plasma treatment was performed to introduce hydrophilic groups like –CO and >C=O (carbonyl) that enhanced the wettability of the pristine PVA. With an applied plasma power of 120 W, the most hydrophilic functional groups were detected. Moreover, a plasma treatment time of 5 s was found to be optimal. To verify the capacitive improvement of plasma-treated PVA, an SSC was assembled using C/MnO_2_ as the positive electrode, C/Fe_2_O_3_ as the negative electrode, and PVA-120W5/Na_2_SO_4_ as the gel-polymer electrolyte. The areal specific capacitance of this SSC was 7.89 mF cm^−2^ at a scan rate of 100 mV s^−1^; this was 2.03 times higher than that of the SSC using pristine PVA/Na_2_SO_4_. In addition, PVA-120W5 matrix was immersed into other liquid electrolytes of 1.0 M H_2_SO_4_ and 1.0 M KOH; PVA-120W5/H_2_SO_4_- and PVA-120W5/KOH-based SSCs showed 2.05 and 2.14 times higher specific capacitance than that of the SSC using pristine PVA, respectively. As a result, facile O_2_ plasma treatment functionalized the surface of pristine PVA to improve its wettability and thereby effectively enhanced the specific capacitance of the as-assembled SSC.

## 4. Materials and Methods

### 4.1. Materials

Potassium hydroxide (KOH, 95.0%), sodium sulfate (Na_2_SO_4_, 98.5%), sulfuric acid (H_2_SO_4_, 95.0%), manganese dioxide powder (MnO_2_), and iron (III) oxide powder (Fe_2_O_3_) were purchased from Samchun Chemical Company (Seoul, Republic of Korea). PVA (*M_w_* 89,000–98,000), PVDF (*M_w_*~534,000), 1-methyl-2-pyrrolidinone (NMP, 99.0%), and Lithium hexafluorophosphate solution (1.0 M LiPF_6_ in EC/EMC 3:7, battery grade) were purchased from Sigma-Aldrich Co. (Burlington, MA, USA). Activated carbon was purchased from Tokyo Chemical Industry Co. (Tokyo, Japan). All chemicals were used as received without any additional purification.

### 4.2. Preparation of PVA-Based Gel-Polymer Electrolyte (Pristine PVA)

PVA-based gel-polymer electrolyte (pristine PVA) was prepared using the simple gelation method. PVA powder (3.0 g) was dispersed in 20 mL of DI water and stirred for 5 h while maintaining a temperature of 85 °C. The resulting solution was then drop-casted onto a PMMA plate and allowed to dry slowly at room temperature for 2 h. The resulting pristine PVA was peeled off from the plate. Then, it was cut slightly larger than the positive and negative electrodes to prevent a short when the assembled SSC was connected to a circuit.

### 4.3. Surface Modification of PVA-Based Gel-Polymer Electrolyte Using O_2_ Atmospheric Plasma Treatment

Surface modification of pristine PVA was performed by atmospheric plasma treatment using a commercially available plasma apparatus (MyPL-100P, APP Co., Ltd., Hwaseong, Republic of Korea) to produce the plasma-treated PVA. A plasma curtain was generated by radio frequency (RF, 13.56 MHz) discharge on a cathode ray tube with an argon flow (flow rate = 2.5 L/min) and O_2_ flow (flow rate = 20 sccm) at an applied plasma power of 80–160 W. After the plasma curtain glowed, the pristine PVA placed at a distance of 0.5 cm from the cathode ray tube was exposed to it for 5–25 s. After completing the plasma treatment, the plasma-treated PVA was produced and kept in a fume hood for 3 h before characterization.

### 4.4. Characterization

The morphological structure of the pristine and plasma-treated PVA were examined using field-emission scanning electron microscopy (S-4800, Hitachi, Tokyo, Japan) and OM (BH2-UMA, Olympus, Tokyo, Japan). The molecular structures of the PVA matrices were characterized using an FT-IR instrument (Nicolet iS10, Thermo Fisher Scientific, Waltham, MA, USA). The surface oxidation states of the PVA matrices were investigated using XPS (K-alpha, Thermo Fisher Scientific, Waltham, MA, USA). The hydrophilicity of the plasma-treated PVA matrices was investigated by measuring the angle of the water droplet using a CA analyzer (Phoenix 300, SEO, Suwon, Republic of Korea. The swelling ratios were determined by immersing the pristine and plasma-treated PVAs in aqueous solution, EtOH, and commercial EC/EMC 3:7 (1.0 M LiPF_6_) electrolyte.

### 4.5. Assembly of SSC

The SSC was fabricated by sandwiching the PVA-based gel-polymer electrolyte between the positive and negative electrodes. The active materials for the electrodes were prepared using a simple paste mixing method by dispersing activated carbon (4.0 g) in 30 mL of EtOH and stirring for 6 h. For the positive and negative electrodes, MnO_2_ (1.2 g) and Fe_2_O_3_ (1.0 g) were dispersed in each solution, respectively. The solutions were shuffled using a paste mixer (PDM-300, DAE WHA TECH, Yongin, Republic of Korea) at a rotation speed of 800 rpm and revolution speed of 700 rpm for 2 h. The resulting solutions were then centrifuged at 8500 rpm for 20 min. A washing cycle was repeated three times, and the resulting C/MnO_2_ and C/Fe_2_O_3_ were obtained after drying in an oven at 70 °C overnight. The positive and negative electrodes were fabricated by mixing 2.0 mg of as-prepared active materials (C/MnO_2_ and C/Fe_2_O_3_, respectively) and 0.1 mg of PVDF as a binder with a few drops of *N*-methyl-2-pyrrolidone (NMP), which resulted in a homogeneous paste. This paste was then applied to a stainless-steel mesh (SUS304, 5 × 2 cm^2^) and dried in an oven at 75 °C. The pristine and plasma-treated PVA were immersed in 1.0 M Na_2_SO_4_, 1.0 M H_2_SO_4_, and 1.0 M KOH for 2 h and then dried at room temperature for 2 h. Finally, the SSC was fabricated by sandwiching the PVA-based gel-polymer electrolyte between C/MnO_2_ as the positive electrode and C/Fe_2_O_3_ as the negative electrode.

### 4.6. Electrochemical Measurement

CV measurements and EIS were performed using an electrochemical workstation (ZIVE SP1, WonATech Co., Seoul, Republic of Korea). For comparison, the CVs of SSCs fabricated using pristine and plasma-treated PVA were measured at a scan rate of 100 mV s^−1^. The voltage range of various SSCs was set to 0–1.5 V. CV measurements of the SSCs fabricated using plasma-treated PVA prepared under the optimal conditions were measured by varying the scan rate from 10 to 200 mV s^−1^. The areal capacitances (mF cm^−2^) were calculated from CV curves using the following equation [84]:

(1) $$C=\frac{1}{S\times \nu \times ∆V}\int_{V_{0}}^{V_{0}+∆V}IdV\;(from\;CV\;curves)$$

where C is the areal capacitance; ν, the scan rate; V, the voltage; ∆V, the operating voltage window; and S, the surface area of the electrode.

EIS analysis of the SSC was performed by applying an AC voltage of 10 mV in the frequency range of 10 mHz to 100 kHz.

## Figures and Tables

**Table 1:** Comparison between the PVA-120W5/Na_2_SO_4_ with the other plasma-treated materials in the fields of SCs.

Materials	Target of the Treatment	Plasma Type	Specific Capacitance		Ref
			Without Plasma	With Plasma	
PVA-120W5/Na_2_SO_4_	gel electrolyte	Oxygen atmospheric plasma	3.89 mF cm^−2^ (at 100 mV s^−1^)	7.89 mF cm^−2^	This work
rGO-MnO_x_	active material or electrode	Nitrogen plasma	9.92 mF cm^−2^ (at 2 mV s^−1^)	52.23 mF cm^−2^	[48]
MWCNTs	active material or electrode	Nitrogen plasma	22 F g^−1^ (at 10 mV s^−1^)	55 F g^−1^	[82]
MWNTs	active material or electrode	Oxygen plasma	61.5 F g^−1^ (at 100 mV s^−1^)	238.2 F g^−1^	[83]

## Data Availability

Data are contained within the article.

## Supplementary Materials

The following supporting information can be downloaded at: https://www.mdpi.com/article/10.3390/gels9040351/s1, Figure S1: SEM images of (a) pristine PVA, (b) PVA-80W5, (c) PVA-100W5, (d) PVA-120W5, (e) PVA-140W5, and (f) PVA-160W5. Figure S2: Electrochemical equivalent circuit model for the EIS analysis. Figure S3: Electrochemical impedance spectroscopy (EIS) analysis including the equivalent circuit model for the SSC devices employing (a) pristine PVA, (b) PVA-80W5, (c) PVA-100W5, (d) PVA-120W5, (e) PVA-140W5, and (f) PVA-160W5. Figure S4: EIS analysis including the equivalent circuit model for the SSC devices employing (a) pristine PVA, (b) PVA-120W5, (c) PVA-120W10, (d) PVA-120W15, (e) PVA-120W20, and (f) PVA-120W25. Figure S5: Long-term cycling test of the SCC device employing pristine PVA and PVA-120W5 with the current density of 5 mA cm^−2^; Table S1: Swelling ratios of pristine and plasma-treated PVAs after immersed in aqueous solution (1.0 M Na_2_SO_4_), EtOH, and commercial electrolyte (1.0 M LiPF_6_ in EC/EMC 3:7).
