# Supplementary material for: Facile Enhancement of Electrochemical Performance of Solid-State Supercapacitor via Atmospheric Plasma Treatment on PVA-Based Gel-Polymer Electrolyte
Source: Gels. 2023 Apr 21;9(4):351. doi: 10.3390/gels9040351 (PMC10137675; doi:10.3390/gels9040351)
Supplement: Supplementary file 1 [file gels-09-00351-s001.zip › gels-2345322-supplementary.pdf]

gels

Supplementary Materials

Facile Enhancement of Electrochemical Performance of Solid-State Supercapacitor *via* Atmospheric Plasma Treatment on PVA-based Gel-Polymer Electrolyte

Dong-Hyun Kim [1,†], Suk Jekal [1,†], Chan-Gyo Kim [1], Yeon-Ryong Chu [1], Jungchul Noh [2], Min Sang Kim [1], Neunghi Lee [1], Woo-Jin Song [3,4,5] and Chang-Min Yoon [1,*]

[1] Department of Chemical and Biological Engineering, Hanbat National University, Daejeon 34158, Republic of Korea
[2] McKetta Department of Chemical Engineering and Texas Material Institute, The University of Texas at Austin, Austin, TX, USA
[3] Department of Polymer Science and Engineering, Chungnam National University, Daejeon 34134, Republic of Korea
[4] Department of Chemical Engineering and Applied Chemistry, Chungnam National University, Daejeon 34134, Republic of Korea
[5] Department of Organic Materials Engineering, Chungnam National University, Daejeon 34134, Republic of Korea

† These authors equally contributed.
* Correspondence: cmyoon4321@hanbat.ac.kr; Tel.: +82-42-821-1528, Fax: +82-42-821-1593.

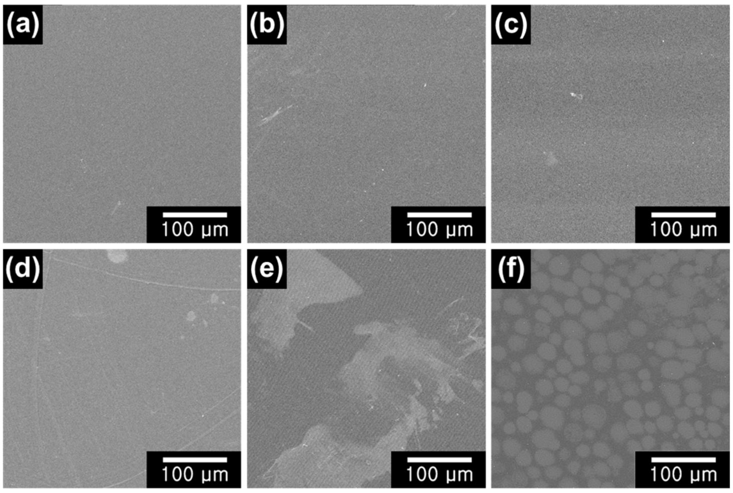

Figure S1. SEM images of (a) pristine PVA, (b) PVA-80W5, (c) PVA-100W5, (d) PVA-120W5, (e) PVA-140W5, and (f) PVA-160W5.

Table S1. Swelling ratios of pristine and plasma-treated PVAs after immersed in aqueous solution (1.0 M Na_2SO_4), EtOH, and commercial electrolyte (1.0 M $LiPF_6$ in EC/EMC 3:7).

**Materials**	**Swelling ratio (M_{wet}/M_{dry})** [a]		
	**1.0 M Na_2SO_4**	**Ethanol**	**1.0 M $LiPF_6$ in EC/EMC 3:7**
pristine PVA	2.3	1.7	1.6
PVA-120W5	3.6	2.6	1.8
PVA-120W10	3.3	2.4	1.6
PVA-120W15	3.2	2.3	1.3
PVA-120W20	3.2	2.3	1.4
PVA-120W25	2.7	1.9	1.1

[a] Swelling ratios were calculated by dividing mass of dried various plasma-treated PVA matrix (M_{dry}) by mass of immersed wet plasma-treated PVA matrix in various solutions for 10 s (M_{wet}).

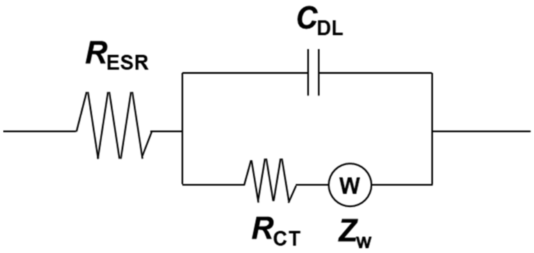

Figure S2. Electrochemical equivalent circuit model for the EIS analysis.

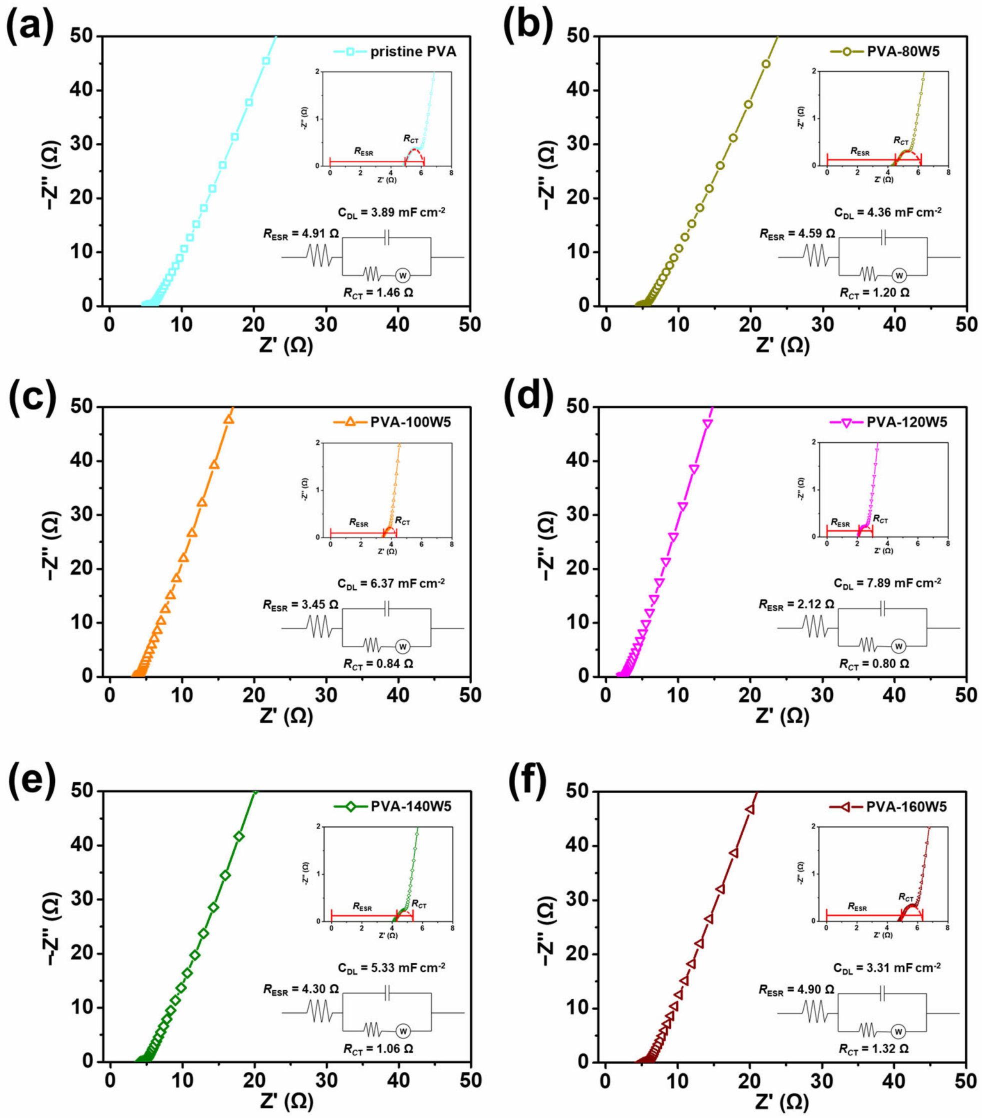

Figure S3. Electrochemical impedance spectroscopy (EIS) analysis including the equivalent circuit model for the SSC devices employing (a) pristine PVA, (b) PVA-80W5, (c) PVA-100W5, (d) PVA-120W5, (e) PVA-140W5, and (f) PVA-160W5.

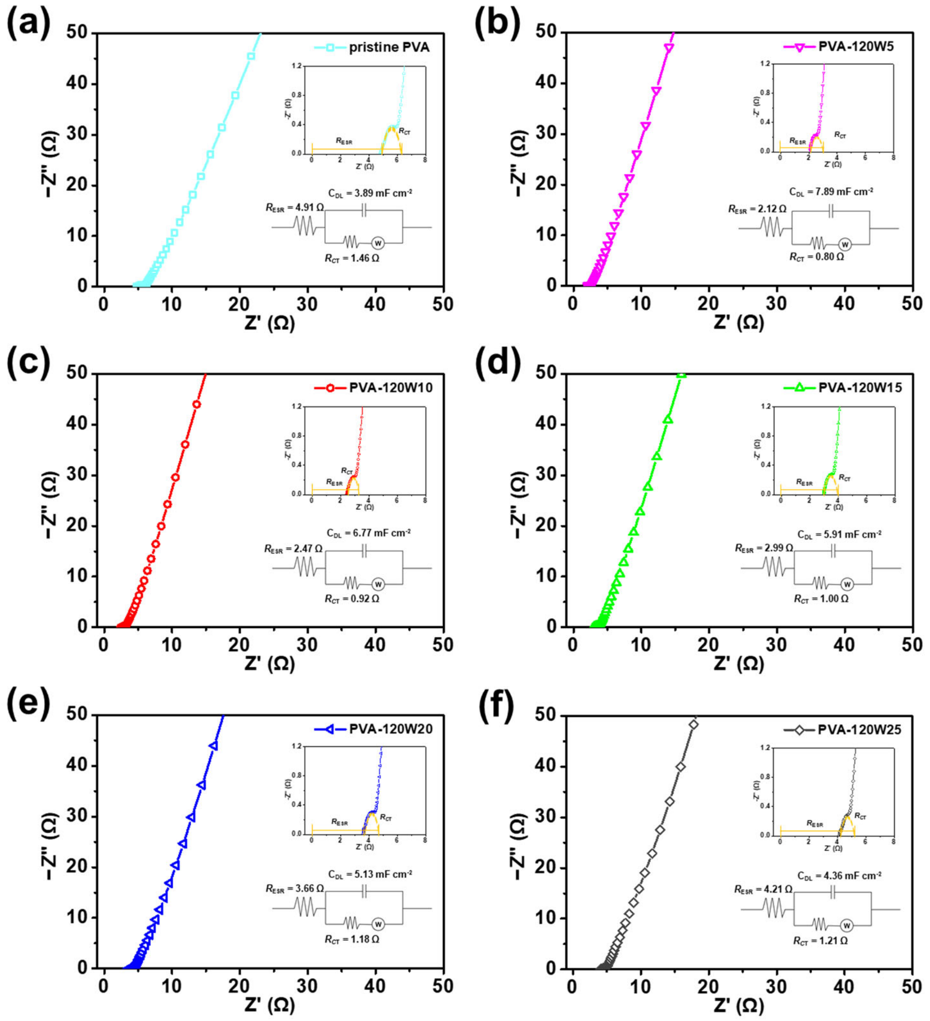

Figure S4. EIS analysis including the equivalent circuit model for the SSC devices employing (a) pristine PVA, (b) PVA-120W5, (c) PVA-120W10, (d) PVA-120W15, (e) PVA-120W20, and (f) PVA-120W25.

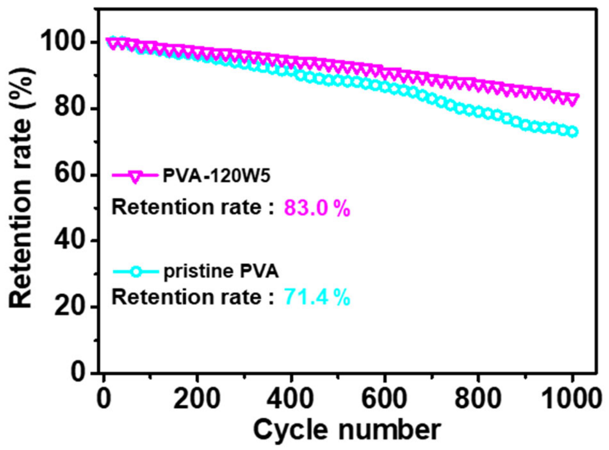

Figure S5. Long-term cycling test of the SCC device employing pristine PVA and PVA-120W5 with the current density of 5 mA cm^{-2}.
